# Usefulness of P‐wave peak time as an electrocardiographic parameter in predicting left ventricular diastolic dysfunction in patients with mitral regurgitation

**DOI:** 10.1111/anec.13000

**Published:** 2022-08-16

**Authors:** Kazuki Ito, Keisuke Miyajima, Tsuyoshi Urushida, Kyoko Unno, Ayako Okazaki, Yasuyo Takashima, Tomoyuki Watanabe, Yoshitaka Kawaguchi, Yasushi Wakabayashi, Yuichiro Maekawa

**Affiliations:** ^1^ Department of Cardiology Seirei Mikatahara General Hospital Hamamatsu‐City, Shizuoka Japan; ^2^ Department of Cardiology Hamamatsu University School of Medicine Hamamatsu‐City, Shizuoka Japan

**Keywords:** atrial pressure, echocardiography, electrocardiography, heart failure, left ventricular diastolic dysfunction, mitral valve regurgitation

## Abstract

**Introduction:**

Conventional Doppler measurements have limitations in predicting left ventricular diastolic dysfunction (LVDD) in patients with mitral regurgitation (MR). Recently, electrocardiographic P‐wave peak time (PWPT) has been proposed as a parameter of detecting LVDD. This study aimed to evaluate the association between PWPT and left ventricular end‐diastolic pressure (LVEDP) in patients with MR.

**Methods:**

We performed echocardiography and cardiac catheterization in 82 patients with moderate or severe MR. We classified patients into two groups: low LVEDP group (L‐LVEDP) (LVEDP <16 mmHg, *n* = 40) and high LVEDP group (H‐LVEDP) (LVEDP ≥16 mmHg, *n* = 42). We evaluated LVDD and PWPT based on echocardiographic and electrocardiographic findings in both groups.

**Results:**

The PWPT in lead II (PWPT_II_) was significantly longer in patients in the H‐LVEDP group than in those in the L‐LVEDP group (67 vs. 47 ms, *p* < .001). Using correlation analysis, LVEDP was positively correlated with PWPT_II_ (*r* = .577, *p* < .001). Using multivariate analysis, PWPT_II_ was found to be an independent predictor of increased LVEDP (95% CI: 0.1030–0.110; *p* < .001). Using receiver operating characteristic (ROC) curve analysis, the optimal cutoff value of PWPT_II_ for predicting elevated LVEDP was 58.9 ms, with a sensitivity of 80.0% and a specificity of 73.8% (area under curve: 0.809, 95% CI: 0.713–0.905).

**Conclusion:**

To the best of our knowledge, this is the first study to assess the effect of a significant valvular disease on PWPT in lead II. These findings suggest that prolonged PWPT_II_ may be an independent predictor of increased LVEDP in patients with moderate or severe MR.

## Graphical abstract

Conventional Doppler measurements have some limitations in predicting left ventricular diastolic dysfunction (LVDD) in patients with mitral regurgitation (MR). Electrocardiographic P‐wave peak time (PWPT) has been proposed as a parameter of LVDD. We evaluated correlation between the PWPT in lead II (PWPT_II_) and left ventricular end‐diastolic pressure (LVEDP) in patients with MR. LVEDP is positively correlated with PWPT_II._

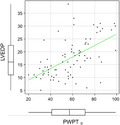

## 1 INTRODUCTION

Left ventricular diastolic dysfunction (LVDD) impairs ventricular filling and increases left ventricular diastolic filling pressure. This abnormality can develop adverse cardiovascular events, such as heart failure and arrhythmia (Wan et al., 2014). Diastolic dysfunction in patients with normal systolic function without HF is a predictor of mortality (Wan et al., 2014). Heart failure with preserved ejection fraction (HFpEF) is a clinical syndrome in patients with current or prior symptoms of heart failure with a left ventricular ejection fraction (LVEF) ≧50%. In addition, most patients with HFpEF display evidence of LVDD (Sharma & Kass, 2014). Worldwide, the proportion of patients with HFpEF is nearly half of all patients with heart failure, and this proportion appears to be increasing (Borlaug & Redfield, 2011).

The natural history of chronic mitral regurgitation (MR) is variable and depends on a combination of factors including regurgitation volume, myocardial status, and etiology of the underlying disorder. In asymptomatic patients with primary severe MR, the rate of progression of symptoms including left ventricular (LV) dysfunction, pulmonary hypertension, or atrial fibrillation (AF) is 30%–40% at 5 years (Bonow, 2013). Recent studies have shown an increased mortality rate in patients with chronic heart failure and secondary MR (Sabbah et al., 1993).

Electrocardiography (ECG) is used in the diagnosis and management of cardiovascular diseases. Although there are no specific ECG findings unique to the presence of diastolic dysfunction, certain ECG findings, such as electrocardiographic left ventricular hypertrophy voltage criteria and P‐wave terminal force (PWTF) in lead V1, are associated with LVDD (Kattel et al., 2016). Recently, electrocardiographic P‐wave peak time (PWPT) has been proposed as a parameter of LVDD (Bayam et al., 2021; Burak, Çağdaş, et al., 2019; Burak, Yesin, et al., 2019; Çağdaş et al., 2017). LVDD leading to increased left ventricular end‐diastolic pressure (LVEDP) causes elevation in left atrial pressure. This atrial overload may appear on the ECG as a prolongation of PWPT. Echocardiography is commonly used to evaluate LVDD. However, conventional Doppler measurements have few limitations in predicting LVDD in patients with MR (Bruch et al., 2004, 2007; Nagueh et al., 2016). Hence, this study aimed to evaluate the association between PWPT and LVEDP in patients with MR.

## 2 METHODS

### 2.1 Study population

This was a retrospective observational study. We included patients with a diagnosis of chronic moderate or severe MR who underwent cardiac catheterization and electrocardiography between September 2013 and April 2020. MR severity was determined using echocardiography. Patients with acute MR, mitral stenosis, persistent or chronic AF, or permanent pacemaker stimulation were excluded from the study. A total of 82 patients were included in the study. Data including previous medical history, medications administered, laboratory investigation reports, and electrocardiography, echocardiography, and cardiac catheterization reports were collected from the patients' electronic medical records. All participants were assessed for the presence of comorbidities such as systemic hypertension, diabetes mellitus, and chronic kidney disease. New York Heart Association (NYHA) classification and diagnosis of heart failure were established according to the Framingham criteria (Mckee et al., 1971). This retrospective observational study was approved by the Ethics Committee of Seirei Mikatahara General Hospital (approval number 20‐74) and conducted in accordance with the guidelines of the Declaration of Helsinki. An opt‐out method on our website was used in the recruitment of participants.

### 2.2 Echocardiographic examination

Echocardiographic examinations were performed using transthoracic echocardiography (Vivid S60, Vivid E9, Vivid S70: General Electric Healthcare Japan Corporation and Affiniti 70: Philips Japan Corporation), according to the standard imaging techniques recommended by the American Society of Echocardiography (ASE) (Lang et al., 2015). LVEF was measured using the parasternal longitudinal view, employing the Teichholz method (normal LV motion). LVEF was measured using the apical 4‐ and 2‐chamber views, employing the modified Simpson method for biplane assessment (LV asynergy had abnormal motion). The Doppler sample volume was placed at the tip of the mitral leaflets to obtain left ventricular inflow waveforms from the apical 4‐chamber view or apical 3‐chamber view. Tissue Doppler imaging was performed with the sample volume placed at the septal corner of the mitral annulus from the apical 4‐chamber view. In this study, the ratio of transmitral E‐wave velocity to transmitral A‐wave velocity (E/A) and transmitral E‐wave velocity to a mitral annular e′ velocity (E/e′) were measured. Left atrial volume (LAV) was measured using the biplane area‐length method (Lang et al., 2005). LAV index (LAVI) was calculated by correcting LAV for body surface area. The peak velocities of tricuspid regurgitation (TR) were derived from transtricuspid continuous wave Doppler recordings. Presence of aortic stenosis (AS), aortic regurgitation (AR), and TR was evaluated using the standard methods recommended by the ASE, and echocardiographic data were collected to assess whether the extent of valvular disease was moderate or severe.

MR severity was determined according to the Japan Circulation Society Guideline (Bonow et al., 2006) that included color Doppler jet area, vena contracta width, effective regurgitant orifice area (EROA), or regurgitant volume (RV). Moderate MR was recognized if color Doppler jet area in the left atrium was 20%–40%, vena contracta width was 0.3–0.69 cm, EROA was 0.2–0.39 cm^2^, or RV was 30–59 ml. Severe MR was recognized if color Doppler jet area in the left atrium was >40%, vena contracta width was ≧0.7 cm, EROA was ≧0.4 cm^2^, or RV was ≧60 ml. MR was classified as primary MR when it was caused by intrinsic disease of the mitral leaflets or as secondary MR when it was caused by intrinsic disease of the left ventricle and/or mitral annulus (Bonow et al., 2006).

### 2.3 Cardiac catheterization

LVEDP is considered as the gold standard parameter of LVDD. To measure LVEDP, a 4‐Fr or 5‐Fr pigtail catheter was inserted into the left ventricle via the radial or femoral arteries. Baseline left ventricular peak pressure (systolic left ventricular pressure [LVP]), left ventricular nadir pressure (diastolic LVP), and LVEDP were measured at rest in a steady state. LVEDP was measured at end‐diastole, which was defined electrocardiographically by the onset of the next cycle's QRS wave from LVP tracing recorded with a 40‐mmHg scale. We classified the patients into two groups: the low LVEDP (L‐LVEDP) group (LVEDP <16 mmHg) and the high LVEDP (H‐LVEDP) group (LVEDP ≥16 mmHg). Aortic pressure was measured with a 4‐Fr catheter or 5‐Fr pigtail catheter via the radial artery or femoral artery. Pulmonary capillary wedge pressure (PCWP), pulmonary arterial pressure (PAP), right ventricular pressure (RVP), and right atrial pressure (RAP) were measured using a 7‐Fr Swan‐Ganz catheter via the jugular or femoral vein.

### 2.4 Electrocardiographic analysis

**FIGURE 1:**
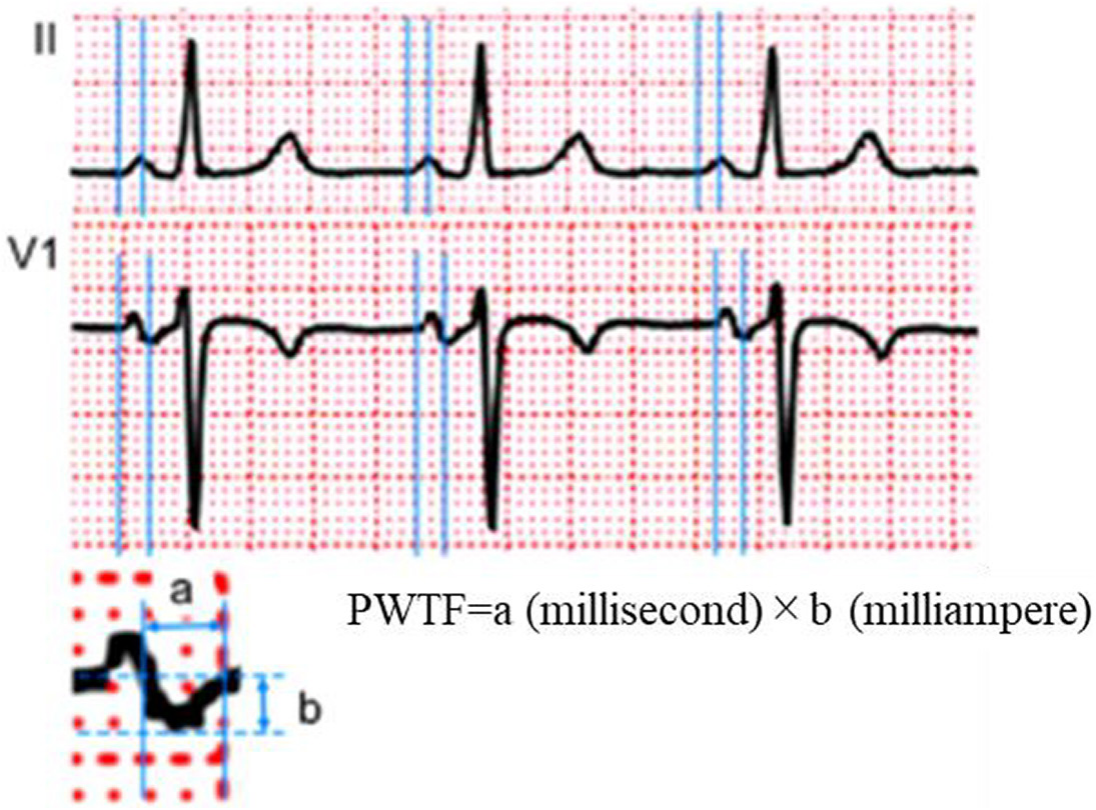
Electrogram showing measurement of P‐wave peak time (PWPT) in lead II. PWPT was defined as the duration between the beginning and the peak of the P wave. In case of biphasic or negative P wave in lead V1, the beginning of the P wave to the nadir of the negative deflection is measured. The P‐wave terminal force is calculated by multiplying the amplitude (mA) and time (millisecond) of the terminal negative component of P wave in lead V1.

A 12‐lead high‐resolution surface ECG was obtained using an electrocardiograph (ECG‐1550, ECG‐2550, STS‐2100; Nihon Koden Corporation) in all participants in the supine position with a filter range of 100 Hz, paper speed of 25 mm/s, and amplitude of 10 mm/mV. All measurements were performed by two independent cardiologists, blinded to the patients' clinical information. All patients breathed freely but were not allowed to speak during the ECG recordings. Measurements were performed using a magnifying lens. The QRS duration was defined as the interval from the start of the QRS complex until the J point. The beginning of the P wave was defined as the point at which the initial deflection of the P wave crossed the isoelectric line, and the end of the P wave was defined as the point at which the final deflection of the P wave crossed the isoelectric line. We measured maximal P‐wave duration time, minimal P‐wave duration time, and average P‐wave duration time in each case. PWPT was defined as the duration between the beginning and the peak of the P wave. In the biphasic P‐wave morphology, which was defined as a P wave with positive and negative deflections, or negative P‐wave morphology, PWPT was measured from the beginning of the P wave to the nadir of the negative deflection. In case of the biphasic or negative P‐wave morphology in lead V1, PWTF was calculated by multiplying the amplitude (milliampere) and time (millisecond) of the terminal negative component of the P wave in lead V1 (Figure 1). All durations were calculated in milliseconds and the mean values were calculated from three or more consecutive beats in each lead.

### 2.5 Statistical analyses

Easy R (EZR) version 1.54 (Division of Hematology, Saitama Medical Center, Jichi Medical University) was used to analyze the data (Kanda, 2013). The Kolmogorov–Smirnov test was used to test for normality. Continuous variables with normal distribution were presented as the mean ± standard deviation. Categorical variables were presented as numbers and percentages (%). Student's *t*‐test was used to compare continuous variables between the two independent groups. Categorical data were compared using Fisher's exact test. Statistical significance was defined as a *p*‐value of <.05. The Pearson correlation coefficient was used to assess correlations between continuous variables and normal distribution. Multivariate logistic regression analyses were performed to identify the independent predictors of the H‐LVEDP group using the variables that showed an association with increased LVEDP in the univariate logistic regression analysis. Receiver operating characteristic (ROC) curve analysis was used to calculate the PWPT value that predicted the H‐LVEDP group with the highest specificity and sensitivity.

## 3 RESULTS

**FIGURE 2:**
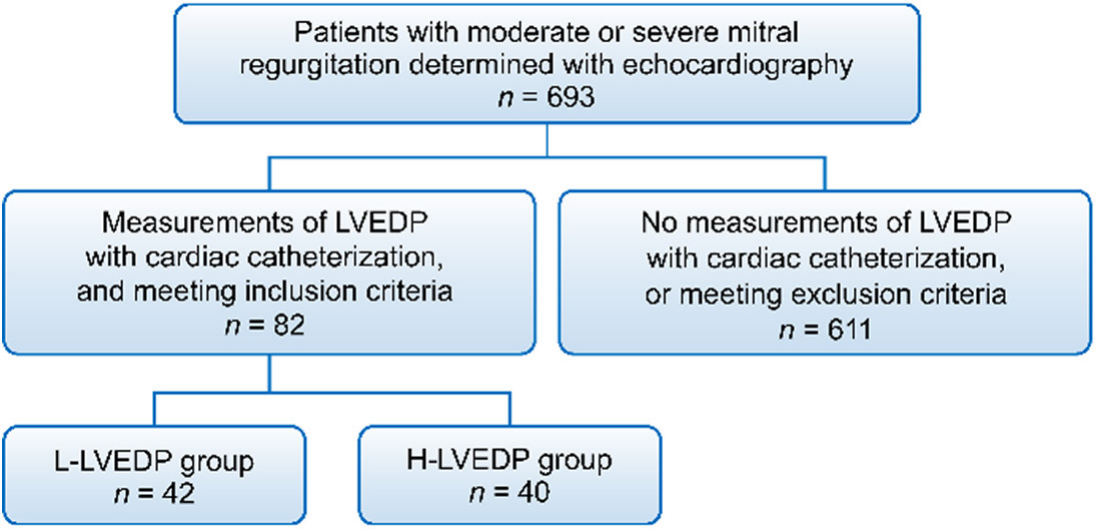
Enrollment of the study participants

**FIGURE 3:**
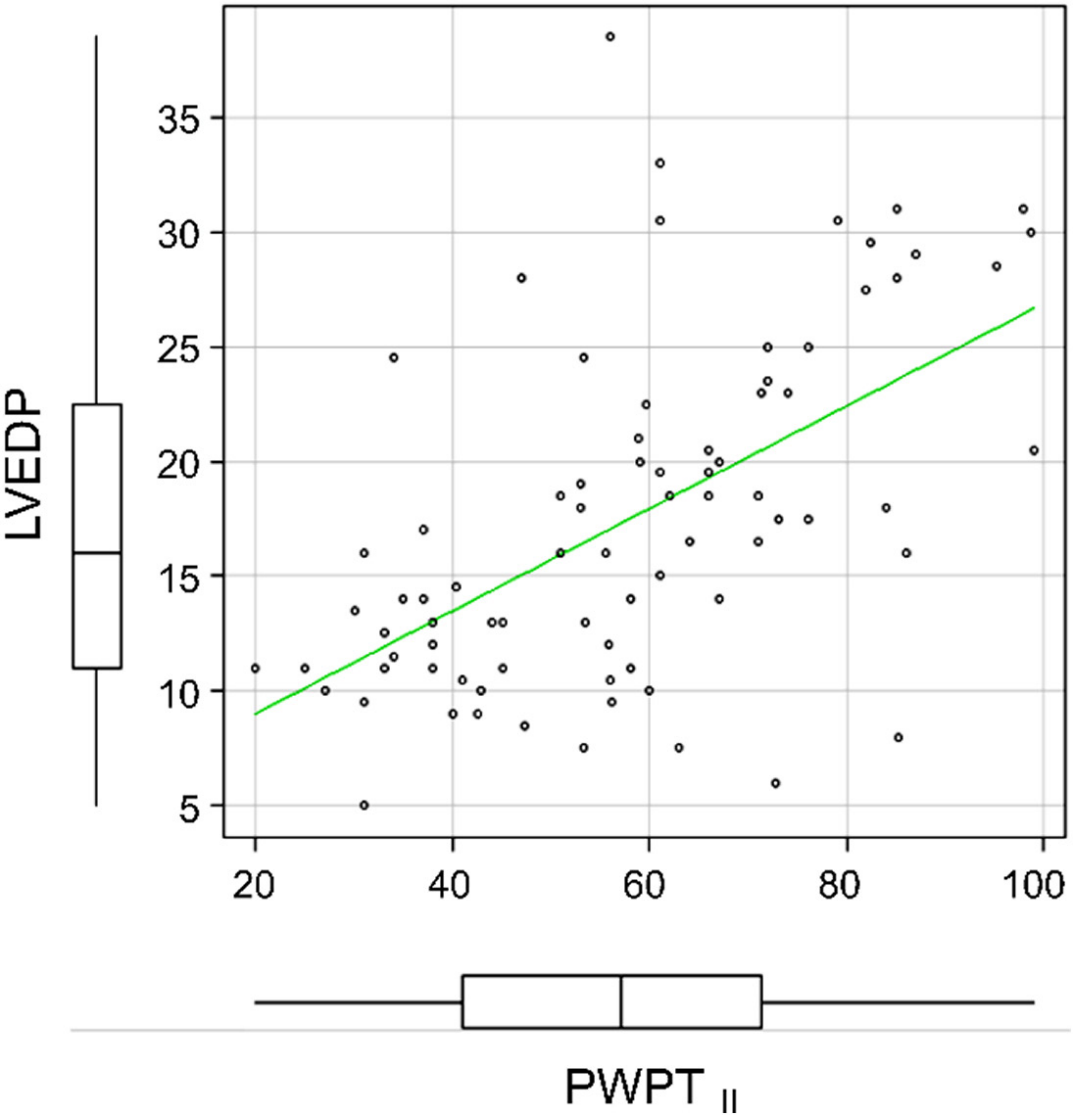
Correlation analysis between the PWPTII and LVEDP. LVEDP is positively correlated with PWPTII (*r* = .577, *p* < .01). LVEDP, left ventricular end‐diastolic pressure; PWPT_II_, P‐wave peak time in lead II; PWPT_V1_, P‐wave peak time in lead V1; PWTF, P‐wave terminal force

**FIGURE 4:**
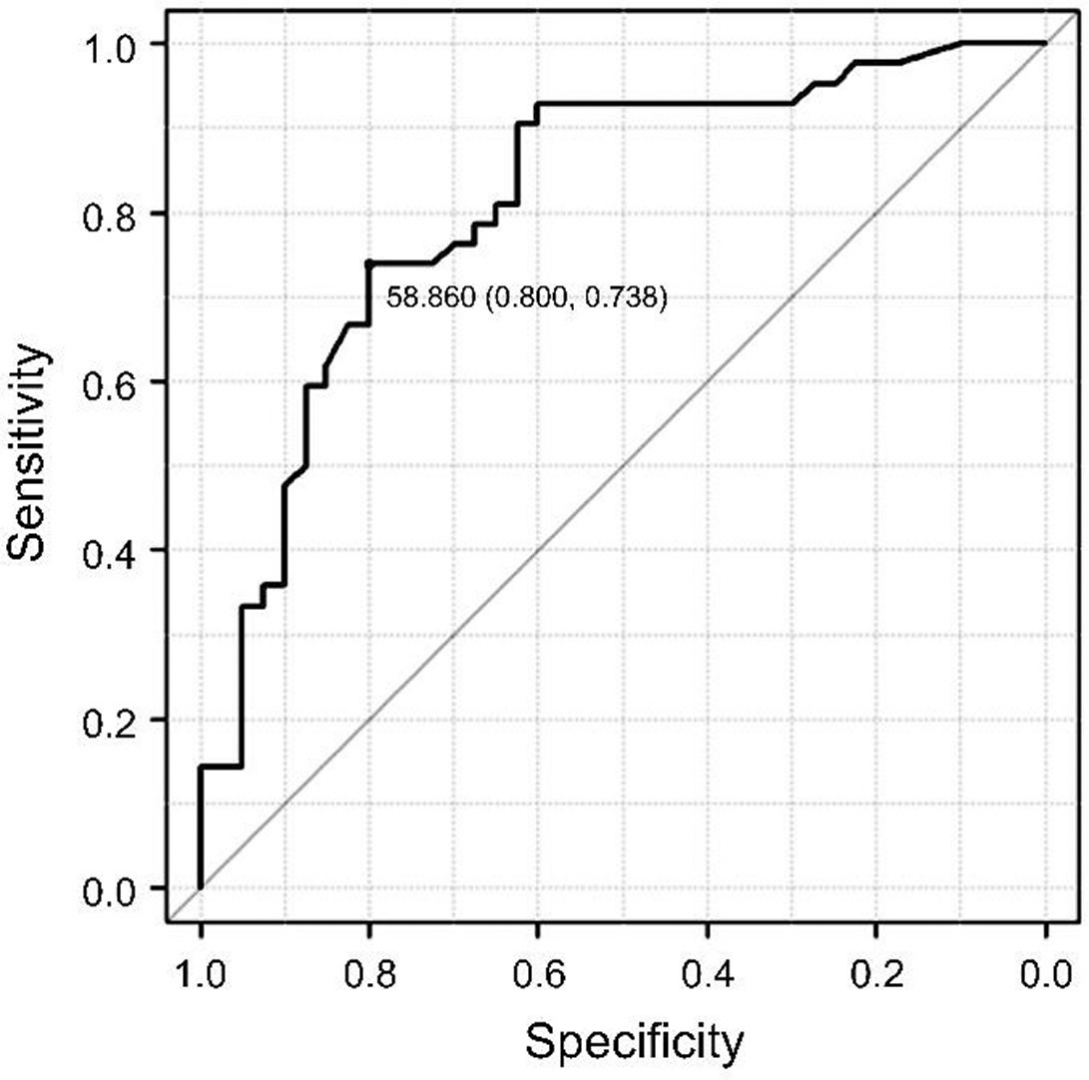
Receiver operating characteristic curve analysis is performed to determine the optimal cutoff value of the PWPTII for prediction of the H‐LVEDP group. The optimal cutoff value of PWPTII for predicting the H‐LVEDP group is 58.9 ms, the specificity for predicting the H‐LVEDP group is 79.5%, and the sensitivity is 73.8%. LVEDP, left ventricular end‐diastolic pressure; PWPT_II_, P‐wave peak time in lead II; PWPT_V1_, P‐wave peak time in lead V1; PWTF, P‐wave terminal force

The number of patients with moderate or severe MR as determined by echocardiography was 693. The number of patients who had no measurements of LVEDP by cardiac catheterization or met the exclusion criteria was 611. This study population consisted of 82 patients with moderate or severe MR who underwent cardiac catheterization for LVEDP measurement and met the inclusion criteria. The numbers of patients in the L‐LVEDP and H‐LVEDP groups were 42 and 40, respectively (Figure 2). The demographic characteristics, laboratory data, and medications of the study groups are shown in Table 1. There was no significant difference between the groups in terms of age, sex, comorbidities, laboratory data, and medication used except the body mass index (20.4 ± 3.4 vs. 21.9 ± 3.6 kg/m^2^, *p* = .05). The cardiac catheterization findings are presented in Table 2. The end‐systolic volume in the H‐LVEDP group was higher than that in the L‐LVEDP group (88 ± 62 vs. 65 ± 56 ml, *p* = .01). Systolic LVP, systolic aortic pressure, diastolic aortic pressure, mean aortic pressure, A‐wave pressure in the PCWP, V‐wave pressure in the PCWP, mean PCWP, systolic PAP, diastolic PAP, mean PAP, systolic RVP, RVEDP, and A‐wave in the RAP were found to be significantly higher in the H‐LVEDP group than those in the L‐LVEDP group (*p* < .05 in each case). Echocardiographic findings are presented in Table 3. LVEF was 59 ± 16% in the L‐LVEDP group and 57 ± 18% in the H‐LVEDP group (*p* = .52). There was no significant difference between the two groups in terms of MR severity, primary MR, secondary MR, AS, AR, and TR except of the number of patients with mitral valve prolapse (25 vs. 17, *p* = .05). Left atrial diameter (LAD), LAVI, E/A ratio, septal e′ velocity, E/e′ ratio, and TR peak velocity were similar between the two groups (*p* > .05 in each case). Table 4 shows electrocardiographic findings. Although there was no significant difference between the two groups in terms of QRS duration, maximal P‐wave duration, minimal P‐wave duration, average P‐wave duration, PWTF in lead V1, and PWPT_V1_ (*p* > .05), PWPT_II_ in the H‐LVEDP group was significantly longer than that in the L‐LVEDP group (67 ± 17 vs. 47 ± 16 ms, *p* < .001). Using the correlation analysis, A‐wave pressure in the PCWP, V‐wave pressure in the PCWP, mean PCWP, and LVEDP were positively correlated with PWPT_II_ (*r* = .500, *r* = .438, *r* = .404, and *r* = .577, respectively; *p* < .001; Table 5 and Figure 3). On the contrary, BNP, QRS duration, maximal P‐wave duration, minimal P‐wave duration, average P‐wave duration, PWTF in lead V1, PWPT_V1_, LAD, LAVI, E/A, septal e′ velocity, E/e′, and TR peak velocity were not correlated with LVEDP. The mean PAP was positively correlated with TR peak velocity (*r* = .501, *p* < .001). The mean PAP and mean PCWP showed mild positive correlation with PWTF in lead V1 (*r* = .373, *r* = .258, respectively, *p* < .05). Univariate logistic regression analyses were performed to determine the clinical variables that associated with increased LVEDP. The clinical variables included body mass index, hemodialysis, paroxysmal AF, moderate or severe AS, LAD, E/e′ ratio, systolic aortic pressure, mean PCWP, average P‐wave duration, PWTF in lead V1, PWPT_II_, and PWPT_V1_. Multivariate logistic regression analyses were performed for those variable (systolic aortic pressure, mean PCWP, and PWPT_II_) that showed significant association with H‐LVEDP. Using multivariate analyses, systolic aortic pressure and PWPT_II_ were found to be independent predictors of H‐LVEDP (OR 1.030; 95% CI: 1.000–1.060; *p* < .05, OR 1.050; 95% CI: 1.010–1.090; *p* < .05, respectively; Table 6). Using ROC curve analysis, the optimal cutoff value of PWPT_II_ for prediction of H‐LVEDP group was 58.9 ms, with a specificity of 79.5% and a sensitivity of 73.8% (area under curve: 0.804, 95% CI: 0.706–0.902; Figure 4).

**TABLE 1:** Baseline characteristics of the patients stratified according to LVEDP

	L‐LVEDP	H‐LVEDP	*p*‐value
	*n* = 40	*n* = 42	
Age, years	72 ± 12	68 ± 13	.16
Female gender, *n* (%)	22 (55.0)	22 (48.9)	.83
Body mass index, kg/m^2^	20.4 ± 3.4	21.9 ± 3.6	.05
Past history/basic heart disease			
Hypertension, *n* (%)	16 (40.0)	24 (53.3)	.13
Diabetes mellitus, *n* (%)	7 (17.5)	8 (17.8)	1.00
Dyslipidemia, *n* (%)	11 (27.5)	19 (42.2)	.11
Chronic kidney disease, *n* (%)	14 (35.0)	18 (40.0)	.50
Hemodialysis, *n* (%)	1 (2.5)	5 (11.0)	.11
Old myocardial infarction, *n* (%)	7 (17.5)	7 (15.6)	1.00
Paroxysmal atrial fibrillation, *n* (%)	7 (17.5)	2 (4.4)	.08
Heart failure, *n* (%)	29 (72.5)	29 (69.0)	.81
NYHA class I, *n* (%)	6 (15.0)	14 (33.3)	.07
NYHA class II, *n* (%)	14 (35.0)	10 (23.8)	.33
NYHA class III, *n* (%)	14 (35.0)	11 (26.2)	.47
NYHA class IV, *n* (%)	6 (15.0)	7 (16.7)	1.00
Laboratory data			
Hemoglobin, g/dl	12.3 ± 2.1	11.9 ± 2.3	.36
Brain natriuretic peptide, pg/ml	470 ± 762	483 ± 644	.94
Hemoglobin A1c, %	6.0 ± 0.8	6.0 ± 1.1	.89
Low‐density lipoprotein cholesterol, mg/dl	107 ± 32	111 ± 36	.65
Creatinine, mg/dl	1.09 ± 1.24	1.86 ± 2.36	.08
Sodium, mEq/L	138 ± 2	139 ± 3	.05
Potassium, mEq/L	4.2 ± 0.6	4.0 ± 0.4	.12
Medication			
Antiplatelet drugs, *n* (%)	12 (30.0)	21 (46.7)	.34
Anticoagulant, *n* (%)	6 (15.0)	3 (6.7)	.31
Beta‐blockers, *n* (%)	11 (27.5)	18 (40.0)	.17
ACE inhibitor/ARB, *n* (%)	11 (27.5)	19 (42.2)	.11
Calcium channel blockers, *n* (%)	6 (15.0)	11 (24.4)	.28
Antiarrhythmic drugs, *n* (%)	5 (12.5)	2 (4.4)	.25

Abbreviations: ACE inhibitor/ARB, angiotensin‐converting enzyme, angiotensin receptor blocker; LVEDP, left ventricular end‐diastolic pressure; NYHA, New York Heart Association.

**TABLE 2:** Cardiac catheterization findings of the patients stratified according to LVEDP

	L‐LVEDP	H‐LVEDP	*p*‐value
	*n* = 40	*n* = 42	
EDV, ml	149 ± 67	174 ± 73	.11
EDVI, ml/m^2^	99 ± 46	114 ± 44	.14
ESV, ml	65 ± 56	88 ± 62	.01
ESVI, ml/m^2^	44 ± 40	57 ± 38	.14
SV, ml	83 ± 33	86 ± 40	.72
SVI, ml/m^2^	54 ± 21	57 ± 25	.56
LVEF, %	59 ± 17	53 ± 19	.10
CO, L/min	4.1 ± 1.6	4.6 ± 1.4	.20
CI, L/min/m^2^	2.7 ± 0.9	3.0 ± 0.7	.28
PVRI, dyne·s·cm^−5^/m^2^	133 ± 110	106 ± 87	.31
SVRI, dyne·s·cm^−5^/m^2^	1199 ± 464	991 ± 502	.11
Aortic pressure			
Systolic pressure, mmHg	114 ± 19	132 ± 25	<.001
Diastolic pressure, mmHg	61 ± 15	68 ± 12	.03
Mean pressure, mmHg	82 ± 15	94 ± 14	<.001
LVP			
Systolic pressure, mmHg	118 ± 13	140 ± 26	<.001
Diastolic pressure, mmHg	−3 ± 6	−2 ± 6	.63
LVEDP, mmHg	10 ± 3	21 ± 6	<.001
PCWP			
A‐wave pressure, mmHg	15 ± 7	22 ± 7	<.001
V‐wave pressure, mmHg	17 ± 10	25 ± 9	<.001
Mean pressure, mmHg	14 ± 8	19 ± 6	.01
PAP			
Systolic pressure, mmHg	33 ± 14	40 ± 11	.03
Diastolic pressure, mmHg	14 ± 6	18 ± 7	.01
Mean pressure, mmHg	21 ± 9	27 ± 8	<.001
RVP			
Systolic pressure, mmHg	35 ± 14	42 ± 10	.03
Diastolic pressure, mmHg	3 ± 3	4 ± 3	.14
RVEDP, mmHg	8 ± 4	11 ± 4	.02
RAP			
A‐wave pressure, mmHg	9 ± 4	11 ± 3	.01
V‐wave pressure, mmHg	7 ± 4	9 ± 3	.08
Mean pressure, mmHg	6 ± 4	8 ± 3	.07
Number of coronary lesions			
No lesions, *n* (%)	27 (67.5)	21 (50.0)	.12
1 lesion, *n* (%)	6 (15.0)	9 (21.4)	.57
2 lesions, *n* (%)	5 (12.5)	6 (14.3)	1.00
3 lesions, *n* (%)	2 (5.0)	6 (14.6)	.26

Abbreviations: CI, cardiac index; CO, cardiac output; EDV, end‐diastolic volume; EDVI, end‐diastolic volume index; ESV, end‐systolic volume; ESVI, end‐systolic volume index; LVEDP, left ventricular end‐diastolic pressure; LVEF, left ventricular ejection fraction; LVP, left ventricular pressure; PAP, pulmonary arterial pressure; PCWP, pulmonary capillary wedge pressure; PVRI, pulmonary vascular resistance index; RAP, right atrium pressure; RVEDP, right ventricular end‐diastolic pressure; RVP, right ventricular pressure; SV, stroke volume; SVI, stroke volume index; SVRI, systemic vascular resistance index.

**TABLE 3:** Echocardiographic findings of the patients stratified according to LVEDP

	L‐LVEDP	H‐LVEDP	*p*‐value
	*n* = 40	*n* = 42	
LVEF, %	59 ± 16	57 ± 18	.52
Moderate MR, *n* (%)	24 (60.0)	33 (78.6)	.09
Severe MR, *n* (%)	16 (40.0)	9 (21.6)	.09
Primary MR, *n* (%)	28 (70.0)	21 (50.0)	.08
Prolapse, *n* (%)	25 (62.5)	17 (40.5)	.05
Secondary MR, *n* (%)	13 (32.5)	21 (50.0)	.12
Ischemic heart disease, *n* (%)	9 (22.5)	13 (31.0)	.46
Non‐ischemic heart disease, *n* (%)	4 (10.0)	9 (21.4)	.23
Moderate or severe AS, *n* (%)	1 (2.5)	6 (14.3)	.11
Moderate or severe AR, *n* (%)	5 (12.5)	7 (15.6)	.76
Moderate or severe TR, *n* (%)	5 (12.5)	3 (6.7)	.48
LAD, mm	42 ± 7	42 ± 7	.80
LAVI, ml/m^2^	59 ± 22	59 ± 15	.98
Peak E‐wave velocity, m/s	1.1 ± 0.4	1.2 ± 0.3	.52
Peak A‐wave velocity, m/s	0.8 ± 0.3	0.9 ± 0.3	.39
E/A ratio	1.5 ± 0.6	1.4 ± 0.5	.86
Septal e′, m/s	0.07 ± 0.03	0.06 ± 0.03	.45
E/e′ ratio	18.0 ± 8.5	19.2 ± 9.8	.62
TR peak velocity, m/s	2.7 ± 0.6	2.8 ± 0.5	.38

Abbreviations: AR, aortic regurgitation; AS, aortic stenosis; LAD, left atrial diameter; LAVI, left atrial volume index; LVEDP, left ventricular end‐diastolic pressure; LVEF, left ventricular ejection fraction; MR, mitral regurgitation; TR, tricuspid regurgitation.

**TABLE 4:** Electrocardiographic findings of the patients stratified according to LVEDP

	L‐LVEDP	H‐LVEDP	*p*‐value
	*n* = 40	*n* = 42	
Heart rate, bpm	72 ± 13	73 ± 13	.63
QRS duration, ms	98 ± 17	104 ± 21	.13
Atrioventricular block, *n* (%)	5 (12.5)	11 (24.4)	.38
Right bundle branch block, *n* (%)	3 (7.5)	2 (4.4)	.67
Left bundle branch block, *n* (%)	0 (0.0)	1 (2.2)	1.00
P‐wave morphology in lead V1			
Negative, *n* (%)	1 (2.5)	2 (4.4)	1.00
Positive, *n* (%)	5 (12.5)	4 (8.9)	.74
Biphasic, *n* (%)	34 (85.0)	36 (80.0)	1.00
Maximal P‐wave duration, ms	118 ± 13	122 ± 17	.20
Minimal P‐wave duration, ms	101 ± 14	102 ± 17	.76
Average P‐wave duration, ms	116 ± 16	119 ± 18	.53
PWTF in lead V1	5.0 ± 3.4	5.0 ± 3.1	.95
PWPT_II_, ms	47 ± 16	67 ± 17	<.001
PWPT_V1_, ms	67 ± 18	65 ± 21	.68

Abbreviations: LVEDP, left ventricular end‐diastolic pressure; PWPT_II_, P‐wave peak time in lead II; PWPT_V1_, P‐wave peak time in lead V1; PWTF, P‐wave terminal force.

**TABLE 5:** Correlation analysis of the laboratory, electrocardiographic, and echocardiographic parameters

	Mean PAP		A‐wave in the PCWP		V‐wave in the PCWP		Mean PCWP		LVEDP	
	*r*‐value	*p*‐value	*r*‐value	*p*‐value	*r*‐value	*p*‐value	*r*‐value	*p*‐value	*r‐*value	*p*‐value
Laboratory data										
BNP	.180	.134	.174	.146	.090	.457	.159	.186	.186	.099
Electrocardiographic findings										
QRS duration	.212	.074	.284	.016	.177	.136	.253	.032	.142	.202
Maximal P‐wave duration	.007	.956	.052	.669	−.015	.901	.055	.649	.069	.540
Minimal P‐wave duration	.070	.563	.116	.335	.097	.423	.141	.241	.024	.829
Average P‐wave duration	−.006	.959	−.035	.773	−.098	.425	−.050	.681	.053	.644
PWTF in lead V1	.373	.002	.232	.065	.125	.325	.258	.040	−.025	.834
PWPT_II_	.275	.019	.500	<.001	.438	<.001	.404	<.001	.592	<.001
PWPT_V1_	.092	.459	.209	.090	.241	.050	.248	.043	−.018	.877
Echocardiographic findings										
LAD	.372	.001	.356	.002	.216	.071	.324	.006	.016	.886
LAVI	.128	.424	.170	.287	.102	.528	.150	.348	.012	.940
E/A ratio	.049	.698	.150	.233	.185	.139	.160	.203	−.028	.817
Septal e′ velocity	−.097	.455	−.158	.221	−.058	.653	−.119	.358	−.016	.893
E/e′ ratio	.296	.019	.341	.007	.388	.002	.363	.004	.018	.883
TR peak velocity	.501	<.001	.305	.024	.205	.133	.264	.052	.176	.176

Abbreviations: BNP, brain natriuretic peptide; LAD, left atrial diameter; LAVI, left atrial volume index; LVEDP, left ventricular end‐diastolic pressure; PAP, pulmonary arterial pressure; PCWP, pulmonary capillary wedge pressure; PWPT_II_, P‐wave peak time in lead II; PWPT_V1_, P‐wave peak time in lead V1; PWTF, P‐wave terminal force; TR, tricuspid regurgitation.

**TABLE 6:** Logistic regression analysis for the prediction of H‐LVEDP group

	Univariable analysis		Multivariable analysis	
	*p*‐value	OR (95% CI)	*p*‐value	OR (95% CI)
Body mass index	.059	1.130 (0.996–1.280)		
Hemodialysis	.138	5.270 (0.588–47.300)		
Paroxysmal atrial fibrillation	.084	0.236 (0.046–1.210)		
Moderate or severe AS	.138	5.270 (0.588–47.300)		
LAD	.795	0.992 (0.931–1.060)		
E/e′ ratio	.616	1.010 (0.962–1.070)		
Systolic aortic pressure	.002	1.040 (1.010–1.060)	.039	1.030 (1.000–1.060)
Mean PCWP	<.001	1.090 (1.020–1.170)	.055	1.090 (0.998–1.180)
Average P‐wave duration	.526	1.010 (0.982–1.040)		
PWTF in lead V1	.951	0.996 (0.862–1.150)		
PWPT_II_	<.001	1.080 (1.040–1.120)	.007	1.050 (1.010–1.090)
PWPT_V1_	.672	0.995 (0.971–1.020)		

Abbreviations: AS, aortic stenosis; CI, confidence interval; LAD, left atrial diameter; LVEDP, left ventricular end‐diastolic pressure; OR, odds ratio; PCWP, pulmonary capillary wedge pressure; PWPT_II_, P‐wave peak time in lead II; PWPT_V1_, P‐wave peak time in lead V1; PWTF, P‐wave terminal force.

## 4 DISCUSSION

There are several findings of this study. First, there were no significant differences in patient background, underlying disease, laboratory results, or medications used between the L‐LVEDP and H‐LVEDP groups, except the body mass index. Second, echocardiographic findings showed no significant differences in LVEF, except the number of patients with mitral valve prolapse. In addition, there was no significant difference between the H‐LVEDP and L‐LVEDP groups in LAD, LAVI, E/A, septal e′ velocity, E/e′, and TR peak velocity. Moreover, there was no correlation between any of the echocardiographic findings and LVEDP. Third, regarding the electrocardiographic findings, the QRS duration, maximal P‐wave duration, minimal P‐wave duration, average P‐wave duration, PWTF in lead V1, and PWPT_V1_ were not significantly different between the H‐LVEDP and L‐LVEDP groups, and there was no correlation with LVEDP. However, PWPT_II_ was significantly prolonged in the H‐LVEDP group and showed a positive correlation with LVEDP. In addition, PWPT_II_ showed a positive correlation with A‐wave pressure, V‐wave pressure, and mean pressure in the PCWP. Fourth, using the ROC analysis, the optimal cutoff value of PWPT_II_ for predicting the H‐LVEDP group was 58.9 ms, the specificity for predicting the H‐LVEDP group was 79.5%, and the sensitivity was 73.8%. In addition, as a result of the logistic regression analysis, systolic aortic pressure and PWPT_II_ were observed to be independent predictors of the H‐LVEDP group.

In general, LVDD is characterized by a slow decline in pressure during isovolumic relaxation, slow left ventricular filling during early diastole, and reduced diastolic compliance during the entire diastole. In addition, the slow left ventricular filling rate results in increased LVP and end‐diastolic pressure (EDP) (Katz & Konstam, 2012). The phase of the P wave in the electrocardiogram is from the left ventricular rapid influx phase to diastole at the onset of left atrial contraction during left ventricular diastole; therefore, the P wave may reflect the left ventricular load in patients with LVDD. According to a previous report, during excessive stretching of the atrium, the membrane resting potential, upstroke velocity, and overshoot of the action potential declined, indicating a decrease in fast sodium current (Penefsky & Hoffman, 1963). This is thought to reduce the conduction velocity of the atrium (Sabine et al., 2003), which may widen the duration of the P wave on the electrocardiogram and prolong PWPT_II_.

In this study, we evaluated whether PWPT_II_ reflected LVDD in patients with moderate or severe MR. We observed that PWPT_II_ showed a positive correlation with LVEDP. There are two possible mechanisms for this. First, it is considered that the end‐diastolic volume and end‐systolic volume increased during the chronic course of MR, and left atrial pressure increased due to an increase in EDP (Apostolidou et al., 2017; Carabello, 1988). This caused atrial enlargement and prolongation of atrial conduction time that resulted in prolongation of PWPT_II_. Second, regurgitant stroke caused the left atrium to expand, which increased compliance of the left atrium (Apostolidou et al., 2017; Carabello, 1988), and prolonged PWPT_II_. According to a previous study of hypertensive patients, PWPT_II_ showed a positive correlation with LVEDP (Burak, Çağdaş, et al., 2019; Burak, Yesin, et al., 2019). In addition, in patients with acute coronary syndrome, it was suggested that PWPT_II_ showed a positive correlation with the complexity of coronary artery lesions, suggesting that the more complex the coronary artery lesions were, the more advanced the LVDD was (Bayam et al., 2021; Burak, Çağdaş, et al., 2019; Burak, Yesin, et al., 2019). These findings have shown that PWPT_II_ may reflect LVDD. Assessment of the impact of significant valvular disease on PWPT_II_ was excluded from all previous studies. However, one of the strengths of the present study is that we examined the effect of valvular disease on PWPT_II_.

Echocardiography is commonly used as a non‐invasive method in the evaluation of left ventricular diastolic function in MR patients (Bruch et al., 2004, 2007). Echocardiographic E/e′ ratio has been shown to correlate with LVEDP in secondary MR with decreased LVEF. However, in primary MR where LVEF is preserved, a previous study has reported that E/e′ ratio did not correlate with LVEDP (Bruch et al., 2004). Moreover, in secondary MR with reduced LVEF, E/e′ was the most accurate parameter among all echocardiographic parameters that predicted high LVEDP (Bruch et al., 2004). In this study, we evaluated patients with relatively preserved LVEF and similar to the previous study, E/e′ ratio did not correlate with LVEDP. Based on its pathophysiological mechanism, E/e′ ratio increases according to mitral regurgitant flow during compensatory MR, and even if LAP is relatively low, E/e′ ratio becomes high. Therefore, E/e′ ratio did not show a significant correlation with LVEDP. In this study, other echocardiographic parameters that indicated diastolic dysfunction did not correlate with LVEDP. One of the reasons may be because this study was a retrospective observational study, and echocardiography was not performed at the same time on all participants. However, the correlation analysis in this study showed that TR peak velocity, which is one of the parameters used to estimate PAP (Galiè et al., 2016), showed a positive correlation with mean PAP. It can be considered that the analyzed echocardiographic parameters were reflected in the hemodynamic findings at the time of EDP measurement.

In this study, multivariate analyses were performed for the factors that predicted the H‐LVEDP group. Among the variables regarding the extracted factors, we selected hemodialysis, paroxysmal AF, moderate or severe AS, and PWPT_II_, which are considered to be associated with elevated LVEDP, or with prolongation of electrocardiographic P wave according to previous studies (Aalaei‐Andabili & Bavry, 2019; Jaroszyński et al., 2018; Nielsen et al., 2015). We observed that PWPT_II_ and systolic aortic pressure were independent predictors of H‐LVEDP. Physiologically, high aortic blood pressure influences the left ventricular wall pressure resulting in left ventricular hypertrophy and increased LVEDP. PWPT_II_ may be a valid index for predicting LVEDP even in patients with moderate or severe MR. On the contrary, PWTF in lead V1 and PWPT_V1_ did not correlate with H‐LVEDP and were not predictors of H‐LVEDP. In lead V1, the P‐wave morphology reflecting atrial stress was observed to be variable in this study; in addition, the measurements of P‐wave duration in lead V1 may be dispersion. Therefore, it is difficult for us to accurately reflect EDP based on P wave in lead V1.

Although high LVEDP may lead to severe LVDD resulting in heart failure (Redfield, 2017; Sharma & Kass, 2014; Zile et al., 2008), the frequency of patients with heart failure and their NYHA class were not significantly different between the L‐LVEDP and H‐LVEDP groups in the present study. The reason for this is that we evaluated patients with moderate or severe MR, and the onset of heart failure may depend not only on LVDD, but also on severity of regurgitant stroke volume. In fact, severe MR tended to be more common in the L‐LVEDP group. It has been shown that the onset of heart failure could not be predicted by LVEDP alone in patients with MR. However, assessment of LVEDP is important to predict progression of MR because the left ventricular end‐systolic volume expands and LVEDP increases when the decompensated period is reached (Nishimura & Schaff, 2013). In this study, cardiac catheterization findings showed that the H‐LVEDP group tended to have higher end‐systolic volume, implying that the H‐LVEDP group included patients with advanced LVDD. Therefore, LVEDP and PWPT_II_ could be factors that affect the onset of heart failure even in MR patients, and PWPT_II_ should be further investigated for their clinical implications in patients with MR.

## 5 LIMITATIONS

This study has several limitations. First, our study had the inherent limitations present in all retrospective observational studies, wherein we could not evaluate prognostic data. Second, echocardiography, electrocardiography, and cardiac catheterization were not performed on the same day and this could be a significant limitation that could affect the results of the study. LVEDP and the grade of MR are affected by the afterload and the patient's volume status. Third, the number of patients included in this study was small. Therefore, future studies with larger patient cohorts are required to overcome these limitations and validate our findings.

## 6 CONCLUSIONS

The present study indicated that prolonged PWPT_II_ could be an independent predictor of elevated LVEDP in patients with moderate or severe MR. This non‐invasive parameter may be useful for evaluating LVDD in patients with MR.

## AUTHOR CONTRIBUTIONS

Kazuki Ito: Responsible for the concept, design, analysis, and writing the manuscript. All authors approved the final manuscript. All authors agreed to their contributions.

## CONFLICT OF INTEREST

The authors declare that there are no conflicts of interest.

## ETHICS STATEMENT

This retrospective observational study was approved by the Ethics Committee of Seirei Mikatahara General Hospital (approval number: 20–74) and conducted in accordance with the guidelines of the Declaration of Helsinki on February 24, 2021.

## PATIENT CONSENT STATEMENT

An opt‐out method on our website was used in the recruitment of participants.

## PERMISSION TO REPRODUCE MATERIAL FROM OTHER SOURCES

Reproduction in any form or by any means, electronic, mechanical, or otherwise, for reasons other than personal use, is strictly prohibited without prior written permission.

## CLINICAL TRIAL REGISTRATION

None.

## Data Availability

The data that support the findings of this study are available on request from the corresponding author. The data are not publicly available due to privacy or ethical restrictions.

## References

[anec13000-bib-0001] Aalaei‐Andabili, S. H. , & Bavry, A. A. (2019). Left ventricular diastolic dysfunction and transcatheter aortic valve replacement outcomes: A review. Cardiology and Therapy, 8, 21–28. 10.1007/s40119-019-0134-5 30847743 PMC6525224

[anec13000-bib-0002] Apostolidou, E. , Maslow, A. D. , & Poppas, A. (2017). Primary mitral valve regurgitation: Update and review. Global Cardiology Science & Practice, 2017, e201703. 10.21542/gcsp.2017.3 31139637 PMC6516795

[anec13000-bib-0003] Bayam, E. , Yıldırım, E. , Kalçık, M. , Karaduman, A. , Kalkan, S. , Güner, A. , Küp, A. , Kahyaoğlu, M. , Yılmaz, Y. , Selcuk, M. , & Uyan, C. (2021). Relationship between P wave peak time and coronary artery disease severity in non‐ST elevation acute coronary syndrome. Herz, 46, 188–194. 10.1007/s00059-019-04859-1 31578616

[anec13000-bib-0004] Bonow, R. O. (2013). Chronic mitral regurgitation and aortic regurgitation: Have indications for surgery changed? Journal of the American College of Cardiology, 61, 693–701. 10.1016/j.jacc.2012.08.1025 23265342

[anec13000-bib-0005] Bonow, R. O. , Carabello, B. A. , Chatterjee, K. , de Leon Jr. A. C. , Faxon, D. P. , Freed, M. D. , Gaasch, W. H. , Lytle, B. W. , Nishimura, R. A. , O’Gara, P. T. , O’Rourke, R. A. , Otto, C. M. , Shah, P. M. , & Shanewise, J. S. (2006). ACC/AHA 2006 guidelines for the management of patients with valvular heart disease: Executive summary. Journal of the American College of Cardiology, 48, e1–e148. 10.1161/circulationaha.106.177303 16875962

[anec13000-bib-0006] Borlaug, B. A. , & Redfield, M. M. (2011). Diastolic and systolic heart failure are distinct phenotypes within the heart failure spectrum. Circulation, 123, 2006–2014. 10.1161/circulationaha.110.954388 21555723 PMC3420141

[anec13000-bib-0007] Bruch, C. , Klem, I. , Breithardt, G. , Wichter, T. , & Gradaus, R. (2007). Diagnostic usefulness and prognostic implications of the mitral E/E' ratio in patients with heart failure and severe secondary mitral regurgitation. American Journal of Cardiology, 100, 860–865. 10.1016/j.amjcard.2007.03.108 17719334

[anec13000-bib-0008] Bruch, C. , Stypmann, J. , Gradaus, R. , Breithardt, G. , & Wichter, T. (2004). Usefulness of tissue Doppler imaging for estimation of filling pressures in patients with primary or secondary pure mitral regurgitation. American Journal of Cardiology, 93, 324–328. 10.1016/j.amjcard.2003.10.012 14759382

[anec13000-bib-0009] Burak, C. , Çağdaş, M. , Rencüzoğulları, I. , Karabağ, Y. , Artaç, I. , Yesin, M. , Çınar, T. , Yıldız, I. , Suleymanoglu, M. , & Tanboğa, H. I. (2019). Association of P wave peak time with left ventricular end‐diastolic pressure in patients with hypertension. Journal of Clinical Hypertension, 21, 608–615. 10.1111/jch.13530 30950573 PMC8030458

[anec13000-bib-0010] Burak, C. , Yesin, M. , Tanık, V. O. , Çağdaş, M. , Rencüzoğulları, İ. , Karabağ, Y. , Hamideyin, Ş. , İliş, D. , Çınar, T. , Altıntaş, B. , & Baysal, E. (2019). Prolonged P wave peak time is associated with the severity of coronary artery disease in patients with non‐ST segment elevation myocardial infarction. Journal of Electrocardiology, 55, 138–143. 10.1016/j.jelectrocard.2019.05.015 31185366

[anec13000-bib-0011] Çağdaş, M. , Karakoyun, S. , Rencüzoğulları, İ. , Karabağ, Y. , Yesin, M. , Gürsoy, M. O. , Artaç, İ. , İliş, D. , Efe, S. Ç. , Taşar, O. , & Karaca, G. (2017). P wave peak time; a novel electrocardiographic parameter in the assessment of coronary no‐reflow. Journal of Electrocardiology, 50, 584–590. 10.1016/j.jelectrocard.2017.06.010 28623012

[anec13000-bib-0012] Carabello, B. A. (1988). Mitral regurgitation: Basic pathophysiologic principles. Modern Concepts of Cardiovascular Diseases, 57, 53–58.

[anec13000-bib-0014] Galiè, N. , Humbert, M. , Vachiery, J. L. , Gibbs, S. , Lang, I. , Torbicki, A. , Simonneau, G. , Peacock, A. , Noordegraaf, A. V. , Beghetti, M. , Ghofrani, A. , Sanchez, M. A. G. , Hansmann, G. , Klepetko, W. , Lancellotti, P. , Matucci, M. , McDonagh, T. , Pierard, L. A. , Trindade, P. T. , … Hoeper, M. (2016). 2015 ESC/ERS guidelines for the diagnosis and treatment of pulmonary hypertension: The Joint Task Force for the Diagnosis and Treatment of Pulmonary Hypertension of the European Society of Cardiology (ESC) and the European Respiratory Society (ERS): Endorsed by: Association for European Paediatric and Congenital Cardiology (AEPC), International Society for Heart and Lung Transplantation (ISHLT). European Heart Journal, 37, 67–119. 10.1093/eurheartj/ehv317 26320113

[anec13000-bib-0015] Jaroszyński, A. , Jaroszyńska, A. , Dąbrowski, W. , Zaborowski, T. , Stepulak, A. , Iłżecki, M. , & Zubilewicz, T. (2018). Factors influencing P terminal force in lead V1 of the ECG in hemodialysis patients. Archives of Medical Science, 14, 257–264. 10.5114/aoms.2017.65926 29593797 PMC5868674

[anec13000-bib-0016] Kanda, Y. (2013). Investigation of the freely available easy‐to‐use software ‘EZR’ for medical statistics. Bone Marrow Transplantation, 48, 452–458. 10.1038/bmt.2012.244 23208313 PMC3590441

[anec13000-bib-0017] Kattel, S. , Memon, S. , Saito, K. , Narula, J. , & Saito, Y. (2016). An effect of left ventricular hypertrophy on mild‐to‐moderate left ventricular diastolic dysfunction. Hellenic Journal of Cardiology, 57, 92–98. 10.1016/j.hjc.2016.03.004 27445022

[anec13000-bib-0018] Katz, A. M. , & Konstam, M. A. (2012). Heart failure: Pathophysiology, molecular biology, and clinical management (2nd ed.). Lippincott Wiliams & Wilkins.

[anec13000-bib-0019] Lang, R. M. , Badano, L. P. , Mor‐Avi, V. , Afilalo, J. , Armstrong, A. , Ernande, L. , Flachskampf, F. A. , Foster, E. , Goldstein, S. A. , Kuznetsova, T. , Lancellotti, P. , Muraru, D. , Picard, M. H. , Rietzschel, E. R. , Rudski, L. , Spencer, K. T. , Tsang, W. , & Voigt, J. U. (2015). Recommendations for cardiac chamber quantification by echocardiography in adults: An update from the American Society of Echocardiography and the European Association of Cardiovascular Imaging. European Heart Journal Cardiovascular Imaging, 16, 233–271. 10.1093/ehjci/jev014 25712077

[anec13000-bib-0020] Lang, R. M. , Bierig, M. , Devereux, R. B. , Flachskampf, F. A. , Foster, E. , Pellikka, P. A. , Picard, M. H. , Roman, M. J. , Seward, J. , Shanewise, J. S. , Solomon, S. D. , Spencer, K. T. , Sutton, M. S. J. , & Stewart, W. J. (2005). Recommendations for chamber quantification: A report from the American Society of Echocardiography's Guidelines and Standards Committee and the Chamber Quantification Writing Group, developed in conjunction with the European Association of Echocardiography, a branch of the European Society of Cardiology. Journal of the American Society of Echocardiography, 18, 1440–1463. 10.1016/j.echo.2005.10.005 16376782

[anec13000-bib-0021] Mckee, P. A. , Castelli, W. P. , McNamara, P. M. , & Kannel, W. B. (1971). The natural history of congestive heart failure: The Framingham Heart Study. The New England Journal of Medicine, 285, 1441–1446. 10.1056/nejm197112232852601 5122894

[anec13000-bib-0022] Nagueh, S. F. , Smiseth, O. A. , Appleton, C. P. , Byrd, B. F., III , Dokainish, H. , Edvardsen, T. , Flachskampf, F. A. , Gillebert, T. C. , Klein, A. L. , Lancellotti, P. , Marino, P. , Oh, J. K. , Popescu, B. A. , & Waggoner, A. D. (2016). Recommendations for the evaluation of left ventricular diastolic function by echocardiography: An update from the American Society of Echocardiography and the European Association of Cardiovascular Imaging. Journal of the American Society of Echocardiography, 29, 277–314. 10.1016/j.echo.2016.01.011 27037982

[anec13000-bib-0023] Nielsen, J. B. , Kühl, J. T. , Pietersen, A. , Graff, C. , Lind, B. , Struijk, J. J. , Olesen, M. S. , Sinner, M. F. , Bachmann, T. N. , Haunsø, S. , Nordestgaard, B. G. , Ellinor, P. T. , Svendsen, J. H. , Kofoed, K. F. , Køber, L. , & Holst, A. G. (2015). P‐wave duration and the risk of atrial fibrillation: Results from the Copenhagen ECG study. Heart Rhythm, 12, 1887–1895. 10.1016/j.hrthm.2015.04.026 25916567

[anec13000-bib-0024] Nishimura, R. A. , & Schaff, H. V. (2013). Mitral regurgitation: Timing of surgery. In Valvular heart disease: A companion to Braunwald's heart disease, Braunwald's Series (pp. 310–325). Elsevier, Saunders. 10.1016/b978-1-4160-5892-2.00017-9

[anec13000-bib-0025] Penefsky, Z. , & Hoffman, B. (1963). Effects of stretch on mechanical and electrical properties of cardiac muscle. American Journal of Physiology, 204, 433–438. 10.1152/ajplegacy.1963.204.3.433

[anec13000-bib-0026] Redfield, M. M. (2017). Heart failure with preserved ejection fraction. The New England Journal of Medicine, 376, 897. 10.1056/nejmc1615918 28249128

[anec13000-bib-0027] Sabbah, H. N. , Rosman, H. , Kono, T. , Alam, M. , Khaja, F. , & Goldstein, S. (1993). On the mechanism of functional mitral regurgitation. The American Journal of Cardiology, 72, 1074–1076. 10.1016/0002-9149(93)90865-a 8213590

[anec13000-bib-0028] Sabine, C. M. , Majidi, M. , van Zandvoort, M. , & Allessie, M. A. (2003). Effects of acute atrial dilation on heterogeneity in conduction in the isolated rabbit heart. Journal of Cardiovascular Electrophysiology, 14, 269–278. 10.1046/j.1540-8167.2003.02280.x 12716109

[anec13000-bib-0029] Sharma, K. , & Kass, D. A. (2014). Heart failure with preserved ejection fraction: Mechanisms, clinical features, and therapies. Circulation Research, 115, 79–96. 10.1161/circresaha.115.302922 24951759 PMC4146618

[anec13000-bib-0030] Wan, S. H. , Vogel, M. W. , & Chen, H. H. (2014). Pre‐clinical diastolic dysfunction. Journal of the American College of Cardiology, 63, 407–416. 10.1016/j.jacc.2013.10.063 24291270 PMC3934927

[anec13000-bib-0031] Zile, M. R. , Bennett, T. D. , St John Sutton, M. , Cho, Y. K. , Adamson, P. B. , Aaron, M. F. , ArandaJr, J. M. , Abraham, W. T. , Smart, F. W. , Stevenson, L. W. , Kueffer, F. J. , & Bourge, R. C. (2008). Transition from chronic compensated to acute decompensated heart failure: Pathophysiological insights obtained from continuous monitoring of intracardiac pressures. Circulation, 118, 1433–1441. 10.1161/circulationaha.108.783910 18794390

